# Summer weather perception and preferences in Powsin Culture Park (Warsaw, Poland)

**DOI:** 10.1007/s00484-023-02455-x

**Published:** 2023-03-27

**Authors:** Katarzyna Rozbicka, Tomasz Rozbicki

**Affiliations:** Department Hydrology, Meteorology and Water Management, Institute of Environmental Engineering, Warsaw University of Life Sciences WULS-SGGW, Warsaw, Poland

**Keywords:** Park, Physiological equivalent temperature, Outdoor thermal comfort, Field survey

## Abstract

Green areas situated inside the city play a very important role for many aspects. Among other, from the social point of view, they have a positive impact on the life of city habitants: directly improve well-being and health, reduce noise, provide opportunities for activity and recreation, improve the tourist attractiveness of the city, etc. The objective of this study was to assess thermal sensations and preferences of people staying outdoors for recreational purposes in the area of the city park during summer 2019, as well as to identify how bioclimate perceptions are modified by personal factors (physical and physiological). In order to determine the optimum thermal zone for recreation and urban tourism during the summer, the regression model for mean thermal preferences (MTPV) every 1 °C in PET value intervals was calculated, and according to this procedure, the preferable spectrum of thermal conditions for tourism and recreation in Warsaw which is related to the PET value range between 27.3 and 31.7 °C. All age groups indicated the highest frequency of neutral thermal sensation vote, which decreased with feeling more extreme thermal conditions. In the case of classification by the gender, the men more than the women indicated the thermal conditions as neutral, “slightly warm,” and “warm.” Studies have shown that women were more sensitive than men to extreme thermal sensations especially “hot” and men more often than women indicated greater acceptance for comfortable and warmer thermal conditions.

**Supplementary Information:**

The online version contains supplementary material available at 10.1007/s00484-023-02455-x.

## Introduction

Contemporary research on the city’s climate focuses mainly on five purposes: evaluation of the urban climate, theoretical base and climate modeling, human thermal comfort, human morbidity and mortality, and the city’s impact on the ecosystem (Hondula et al. 2017). Important elements of urban areas are city green areas. Their importance applies to many aspects of the functioning of urban agglomerations. City green areas change the structure of the hydrological balance of urban areas, making it similar to the balance of natural areas. Due to this change, as well as the change in the structure of the surface, green areas contribute to the cooling effect, which in turn leads to the weakening of the urban heat island phenomenon. This especially applies to city parks, i.e., green areas located within the city. Aram et al. (2019a, 2019b) found that large parks with an area of more than 10 ha lower the temperature by 1.2 °C, and this effect extends to a distance of 350 m from the park boundary. Moreover other hand, from the social point of view, green areas have a positive impact on the life of city habitants: they directly improve well-being and health, reduce noise, provide opportunities for activity and recreation, improve landscape values, often improve the tourist attractiveness of the city, etc.

Research on the importance of urban green areas has been carried out for many years and concerns practically the whole world and all climate zones (Dunnett et al. 2002; Senanayake et al. 2013; Vatseva et al. 2016). Different countries may have a similar or different type of urban green space, depending on the location, geography, climatic zone as well as socio-economic, environmental, and cultural factors of the country (Dunnett et al. 2002). Last years, for Central Europe, were published studies for Warsaw (Lindner-Cendrowska and Błażejczyk 2018; Rozbicka and Rozbicki 2018, 2021); for selected cities in the Czech Republic (Lehnert et al. 2021a, 2021b); for Szeged, Hungary (Kothencz and Blaschke 2017); for Stuttgart, Germany (Keterer and Matzarakis 2014); for Munich, Germany (Zölch et al. 2019); for Western and Southern Europe for selected areas of the Netherlands (Klemm et al. 2015); Florence, Italy (Petralli et al. 2015); for Spain (Aram et al. 2019a, 2019b; Gozalo et al. 2019); and for Lisbon, Portugal (Oliveira and Andrade 2007). For other continents and other climatic zones, studies are known, among others for Kair, Egypt (Fahma and Sharples 2009); for Mendoza in Argentina (Puliafito et al. 2013); for Sidney, Australia (Spagnolo and de Dear 2003); for Wuhan, China (Liu et al. 2017); and for Kuala Lumpur, Malaysia (Ghaffarianhoseini et al. 2019).

An important factor in the research is the impact of various aspects on the perception of thermal conditions, including urban greenery, personal factors, thermal sensations, physical activities, and thermal preferences. In research, Kothencz and Blaschke (2017) showed a relationship between the perception of the surveyed green areas and objective environmental indicators. It was found that subjective assessments and objective data reveal different aspects of the same reality and are fundamental to properly catch the role of urban green spaces in the quality of life. In the studies of Kothencz and Blaschke (2017), Peschardt et al. (2016), and Nordh et al. (2011), they also found that the proportion of urban parks covered with vegetation is a key factor influencing visitor perception and promotes the recreational use of green spaces. It also confirms the formulated hypothesis by these researchers that “Parks with a larger share of green areas are more attractive to visitors.” Kothencz and Blaschke (2017) also point to another important aspect as the surroundings of the park and the perceived disturbances (e.g., density of buildings, aesthetics of buildings around parks), which is crucial in the perception of the attractiveness of city parks (Grahn and Stigsdotter 2010; Peschardt et al. 2016). Whereas results of research conducted on the campus in Tehran (Zafarmandi et al. 2022), Iran showed that more men (95–97%) than women (75–85%) preferred more sun, higher temperature, and less wind during the year. On the other hand, 39% of men and 29% of women, respectively, during the year prefer a neutral and cool thermal sensation which may indicate thermal adaptation. In winter, women prefer milder temperatures, which also proves adaptation to difficult situations, but in summer, there is women’s dissatisfaction of thermal conditions who due to social norms must wear hijab and be fully covered. In the same study, it was shown that increases in air temperature, relative humidity, and solar radiation lead to the increase in thermal sensation while the relation with wind speed is reversed. In another study, Andrade et al. (2011), on the example of Lisbon (Portugal), showed that preferences for different ranges of air temperature depend on the season and are strongly related to wind speed. Most people declared a preference for lower wind speeds in all seasons. The perception of wind is significantly different depending on gender. Women declared a lower level of comfort at higher wind speeds. There was a general decrease in the sensation of discomfort with age.

From the socio-economic point of view, three criteria can be stated as the basis for the classification of green areas in the city (Fratini and Marone 2011; Hansen and Pauleit 2014; Chojnacka 2014; Kłopotowski 2016):

- Availability of the area related to its ownership and spatial separation: with full access, limited access, no available access, or with the permission of the owner.
- Utility functions related to purposes, i.e., separating, insulation, recreational activity and leisure, surrounding the buildings, and agricultural production.
- Method of arranging and equipping the area: designed and implemented comprehensively, created with public participation, developed as a result of lawlessness investment, undeveloped areas.

Taking into account the above criteria, the analyzed Powsin Culture Park is the area with full access, designed and implemented comprehensively for recreational and leisure purposes.

The aim of this study was to assess thermal sensations and preferences of recreationists (i.e., tourists and people staying outdoors for recreational purposes) in the area of the city park, as well as to identify how personal factors (physical or physiological) modify bioclimate perception. To achieve this goal, weather perception surveys with micrometeorological measurements were conducted in the area of the Powsin Culture Park in Warsaw (Poland). The thermal sensations and preferences towards various meteorological elements were examined as well as personal factors (e.g., gender, age, type of activity) influencing the thermal perception of the respondents were identified.

## Materials and methods

### Study area and data

Weather perception studies were conducted with a survey system, collecting information from people staying in the park. Simultaneously with them, in situ micrometeorological measurements were carried out using the Davis mobile weather station. Meteorological measurements were also carried out at the Warszawa-Ursynów station (*ϕ* = 52°09′ N; *λ* = 21°02′ E; *H* = 102 m above sea level) located approximately 5.5 km away of the park and representative for loose urban building.

The research was conducted for 23 days in the summer of 2019 in the period from June 22 to August 31.

Warsaw is located in central Poland, on the Masovian Lowland, covers an area of 517 km^2^, and its population is approximately 1.7 million. The altitude is in the range of 78–121 m asl. According to the Köppen-Geiger climate classification (Peel et al. 2007), it belongs to the humid continental climate zone (Dfb) with cool, cloudy winters and relatively warm summers. The average air temperature in the period 2001–2020 at the Ursynów station ranged from − 1.4 °C in January to 20.5 °C in July. In the summer months, it was 18.2 °C for June, 20.5 °C for July, and 19.8 °C for August, respectively. In terms of thermal conditions, the summer period in 2019 was different from the period 2001–2020. June was the hottest month this summer with the air temperature higher than normal by 4.9 °C. In turn, July was slightly cooler by 0.9 °C, and August was slightly warmer by 1.5 °C compared to the average temperature.

The weather and synoptic situation in the study period of summer 2019 was related to the dominant direction of advection—NE and NW in July and NE, SE, and SW in August. Therefore, July was classified as thermal “normal” and August as “extremely warm” with heat waves also occurring during the days of the survey. In August, the actual insolation was significantly higher (approx. 280 h) than in July. However, precipitation was very low for both months, which amounted to about 30 mm and were classified as “very dry” months, deepening the long-term drought and the risk of fires (Institute of Meteorology and Water Management 2021a, 2021b).

**Fig. 1:**
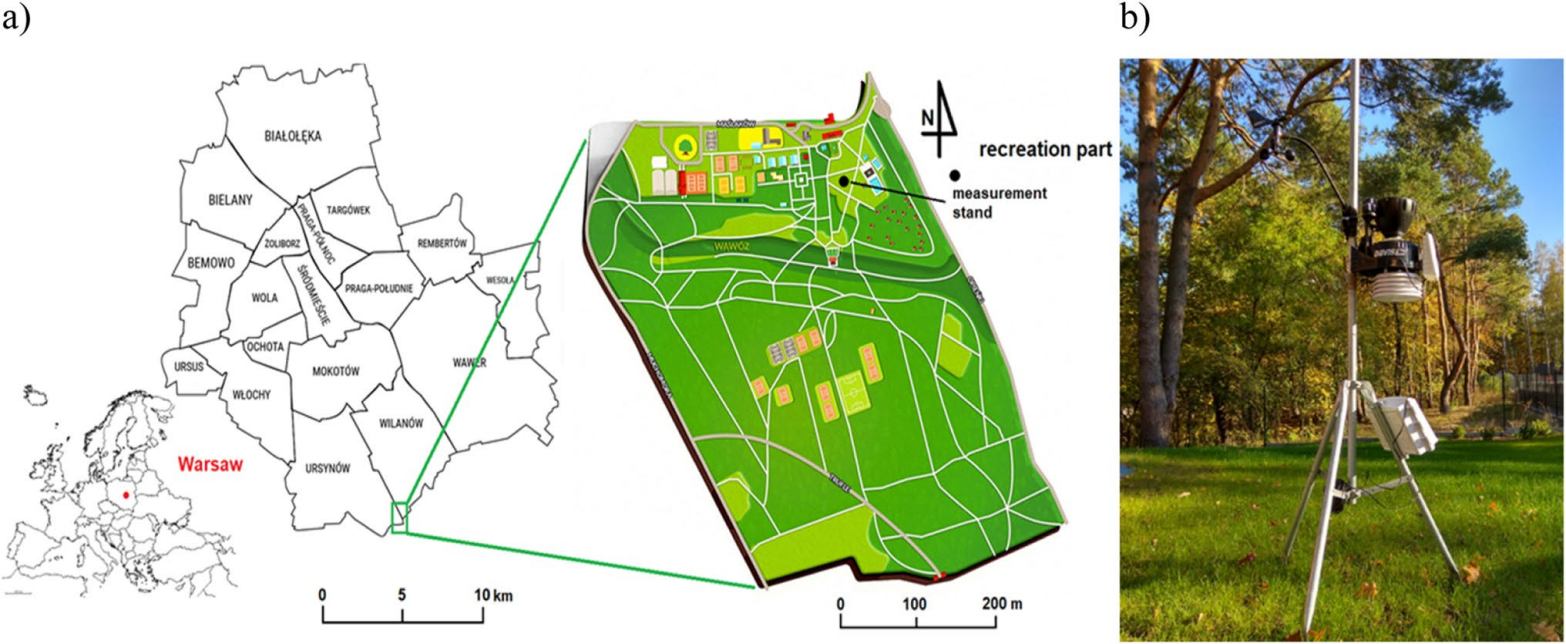
Situated of Warsaw and Powsin Culture Park—**a** research area, **b** mobile weather station

The Powsin Culture Park is situated in the southern part of Warsaw agglomeration, between the Ursynów and Wilanów districts (a detailed location of Warsaw and the park is shown in Fig. 1). It is separated from the city by a large forest complex—Kabacki Forest. The area of the park covers 50 ha, but the area most frequently visited by tourists, where surveys were mainly conducted study area was the part 3.5 ha. The topography is varied, and the gorge spreading from East to West divides the park area into two parts. Fragments of tourist routes run through the Powsin Park crossing the border at the crossroads in the proximity of the clearing. At the beginning, the route is common, but then the routes separate. One of these routes leads east through the recreation and sports areas of the park, while the other turns into the street and reaches the Botanical Garden of the Polish Academy of Sciences. The eastern part of the area has mainly recreational and sports functions, and here there is most of the attractions are located, such as a seasonal swimming pool, a holiday resort, a modern playground, a mini golf field, volleyball and beach volleyball courts, and a sports pavilion with a gym, sauna, bowling alley, and tennis courts. A mobile micrometeorological measurement station was located in an open space in this part of the park. The western part of the park is mainly forest (Culture Park in Powsin 2021). Thanks to its location south of the large districts of Ursynów, Wilanów, and Mokotów, Powsin Culture Park is a place often visited, mainly by the residents of the capital, but not only. In summer, the swimming pool and playgrounds are particularly popular attractions. Access to this site is quick and easy by city bus, car, and bicycle, which in the studied summer period was a frequent form of access.

Meteorological elements were measured by the use of a Davis mobile station (Vantage Pro2 console) that directly connects to the Weather Link Data Logger (# 6510 USB), which was equipped with sensors of air temperature and relative humidity, solar radiation, wind speed and direction, barometric pressure, and a rain collector. Air temperature (Ta in °C), relative humidity (RH in %), and global solar radiation (Kglob in W∙m^−2^) were measured at 1.2 m above the ground level. Wind velocity (v in m∙s^−1^) was measured at 1.6 m height. Meteorological data from sensors were collected every 1 min and averaged every 10 min. Cloudiness data (N8 in octas) came from the Warszawa Okęcie station, located 10 km away from Powsin (Institute of Meteorology and Water Management 2021b). Then, the PET index was determined in RayMan Pro software (Matzarakis et al. 2010). The PET (°C, Physiological Equivalent Temperature) comfort index, derived from the human heat balance model, combines the elements of weather and thermo-physiological (clothing and human activities). It is used to measure the thermal comfort of an individual in a given situation by comparing her physiological responses to those she would have in the reference environment, for example, an office in which she feels comfortable. It is the operative temperature of a reference environment that would cause the same physiological response in the subject as the environment under study, i.e., the same skin temperature and core temperature (Unversite Paris-Saclay 2022).

### Field survey

We conducted interviews of the weather perception and preferences of surveyed people in the research area in the same thermal environment as the measuring weather station as well as Spagnolo and de Dear (2003), Oliveira and Andrade (2007), and Lindner-Cendrowska and Błażejczyk (2018) and determined according to the ISO 10551 (1995) methodology. All survey studies were conducted in accordance with general ethical standards, with preserve full anonymity and voluntariness. The research was conducted mainly in Polish language, and only in a few cases English was used. The response time given in the questionnaires was written, which made it possible to adjust people’s responses to the same current measured weather conditions. The questionnaire included 3 parts and was completed on average for about 2 min. The first part of the questionnaires contained thermal sensations and the preferences of some meteorological elements. Similarly, following previous studies, the seven-point ASHRAE scale was used (Thorsson et al 2004; Lin et al 2011; Bröde et al. 2012 and Krüger et al. 2013) in which the interviewers reported the perception of meteorological conditions, with − 3 corresponds to “cold,” 0 to “neutral,” and + 3 to “hot” thermal sensation. To determine preferences for weather condition, a 3-point McIntyre scale was chosen, where the number 0 means “no change,” and − 1 or + 1 expresses the preferences of reducing or increasing weather elements. The second part of the survey concerned the items of clothing worn by the respondents, their last (30 min) place of time and their physical activity, and the purpose of their arrival at the park. The third part of the survey contained individual data: age, gender, health status, planned duration of staying in this place (number of hours), and district or place of residence. A specimen of the questionnaire used in the research is attached in Appendix 1 (Appendix 1). The total insulation of the garment was calculated according to ISO 9920 (2007).

### Statistical analysis

Student’s *t*-test was used to check did the purpose of staying outdoors have an effect on thermal perception. Regression analysis was performed to state the relationship between the respondent’s thermal sensations and actual meteorological conditions (determined by PET) and some meteorological parameters. For analysis of the relationship between the personal characteristics of the respondents and their thermal sensation votes (TSV) or thermal preference votes (TPV), the correlation and determination coefficients for variables were used. Statistical calculations were made using the Statistica 13.1 program.

The thermal pattern of a population is best represented on the 9-point thermal sensation scale—an approach that is also applied as the Physiological Equivalent Temperature (PET). To describe the thermal sensation of people, the most common studies are based on questionnaire surveys that used the 7-point American Society of Heating, Refrigerating and Air-Conditioning Engineers (ASHRAE) scale. However, the difference between the 7-point scale of ASHRAE and the 9-point scale of PET is the significant disadvantage. Kantor et al. (2012a, b) made an attempt to adapt both scales, not only to obtain thermal sensations category thresholds but also to differentiate the thermal preferences from the thermal sensations of humans. In order to reveal the thermal assessment patterns, the MTSV and MTPV responses were plotted against the PET index. Because subjective thermal assessments can differ greatly among the individuals, even under the same conditions, the procedures of similar studies to decrease the number of individual differences were followed (Kovács et al. 2016).

The calculation of bin-averaged votes entailed the averaging of TSV and TPV values per 1 °C PET interval (bin). Then, regression analyses between the subjects’ mean TSVs/TPVs and the PET bins were performed. The advantage of this method is that it allows for neutral and preferred temperatures to be identified. The neutral PET temperature is derived by setting TSV equal to zero in the mean MTSV vs. PET regression function. Similarity, the preferred temperature is found at the point where the mean TPV vs. PET function intersects the TPO = 0 line (Figs. 5 and 6).

The other advantage of this method is that it allows introducing new thermal sensation category thresholds. In this case, the new thermal sensation thresholds were derived by substituting TSV with − 3.5, − 2.5, − 1.5, …, 3.5 values into the mean MTSV vs. PET regression equation. By definition, the MTSV range of − 0.5 to 0.5 corresponds to the neutral thermal sensation category. Following the same way, the thermal preference ranges were identified. However, while the TSV scale ranges from − 4 to 4, the MTPV scale spans between − 1 and 1 only. In order to be consistent with the previously applied approach, we divided the TPV axis into eight equal parts of 0.25 in length. Here, the − 0.125 to 0.125 TPV range corresponds to the most optimal “preferred” or “comfortable” range (Fig. 7) The procedure developed by Kovács et al. (2016) was applied.

## Results

### Characteristics of the respondents

Field research was conducted in the culture park in Powsin, and 776 surveys were obtained. A total of 50.5% of the respondents were men and 49.5% were women. People aged 30–44 (29.0%) were the most numerous group, next people aged 45–65 (23.2%) and over 65 (23.0%). The clearly smaller groups are people aged 15–29 (13.7%) and children under 15 (11.1%). Groups under 15 and over 65 are usually ignored in thermal comfort studies, but in this study, the group over 65 is a numerous one and characteristic of this place, so it is included in these studies. Usually, all interviewees came to the park for recreation and leisure or tourism. Most of the respondents (85.3%) were outside before reaching the park, and the rest were indoors (at home) or in the car. The dominant activity of the respondents 30 min before the survey was active recreation: walking (32.0%) and cycling (28%) and, slightly less, non-active rest—sitting (28%). Most respondents were residents of Warsaw, and only in very rare cases of respondents came from another city or abroad. They were residents of the nearest districts (surrounding areas) of the park, such as Ursynów, Wilanów, and Mokotów, most often, although there were also residents from further districts such as Southern Praga Pd, Centrum, and Żoliborz (these respondents often came to the park on their bikes). A total of 33.7% of the respondents (17.9% of women and 15.8% of men) gave they suffer from chronic diseases (hypertension, coronary or rheumatic diseases, respiratory system diseases and asthma, etc.). Among women, the most common (48.2%) were respiratory diseases (including asthma) and 32.8% were hypertension, while in men, the most common were hypertension (44.6%) and respiratory diseases (29.8%). A statistically significant relationship (*p* = 0.05) between health status and thermal sensations or preferences was demonstrated, and it decided to include this group of respondents to the sample. Clothing thermal insulation (Icl) is an important factor influencing the thermal sensations of human, especially in the climates where fluctuations of air temperature during the year are considerable. During the hot summer days of the year 2019, clothing of insulation of 0.4 Clo was usually used.

## Biometeorological conditions in the research

The weather conditions during the sampling periods were mostly typical for summer and the district of southern Warsaw, with the exception of a few days in July during which heat waves occurred. The average air temperature was 26.5 °C, with a maximum value of 34.8 °C. The average total solar radiation ranged from 58.0 to 921.0 W∙m^−2^. High values of the standard deviation show significant variability in individual days due to the dynamically changing cloudiness. The average value of the relative humidity was 45.3% and ranged within wide limits from 33.5 to 71.0%, while the recorded wind speed was low with the average value of 1.0 m∙s^−1^, which resulted from the park’s surrounding and the presence of trees (Table 1).

**Table 1:** Characteristics of meteorological elements during field surveys in the Powsin park

	Average	Min	Max	SD
Air temperature (°C)	26.5	17.9	34.8	3.6
Relative humidity (%)	45.3	33.5	71.0	6.9
Wind speed (m·s^−1^)	1.0	0.1	4.7	0.8
Global solar radiation (W·m^−2^)	527.7	58.0	921.0	232.1

**Fig. 2:**
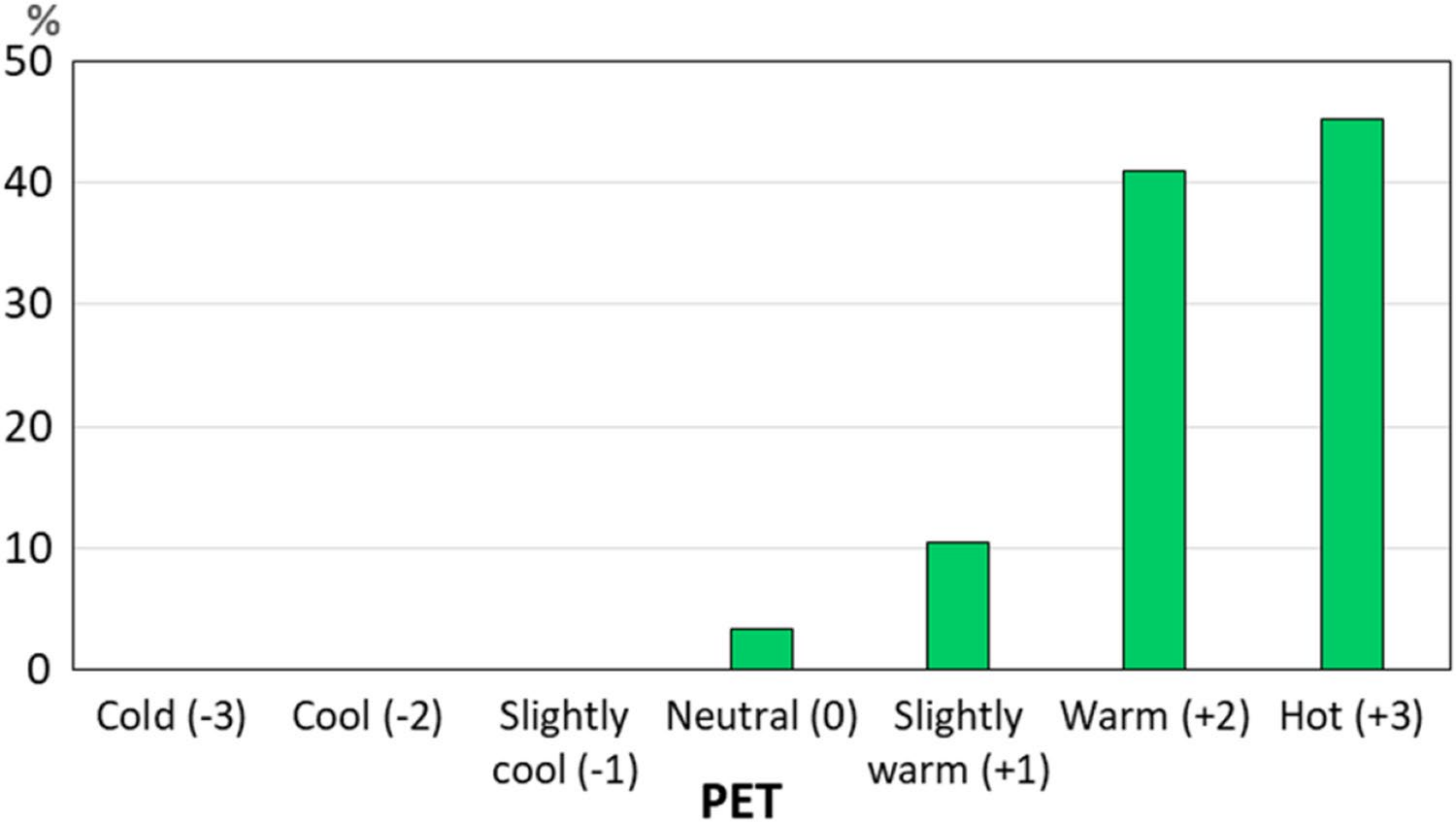
The frequency of thermal sensation (%) in relation to PET ranges. The latter were determined for Central Europe by Matzarakis and Mayer (1996)

During the field study period in the Culture Park in Powsin, PET values varied from 18.6 °C (4th July) to 47.7 °C (26th June), reflecting the thermal sensation from neutral to hot (data not shown). In the analyzed period of summer, the following classes were dominant: “hot” (45.2%) and “warm” (41.0%), which together give the amount 86.2%. Only 10.4% were more pleasant thermal sensations—“slightly warm” and “neutral” (3.4%) (Fig. 2).

**Fig. 3:**
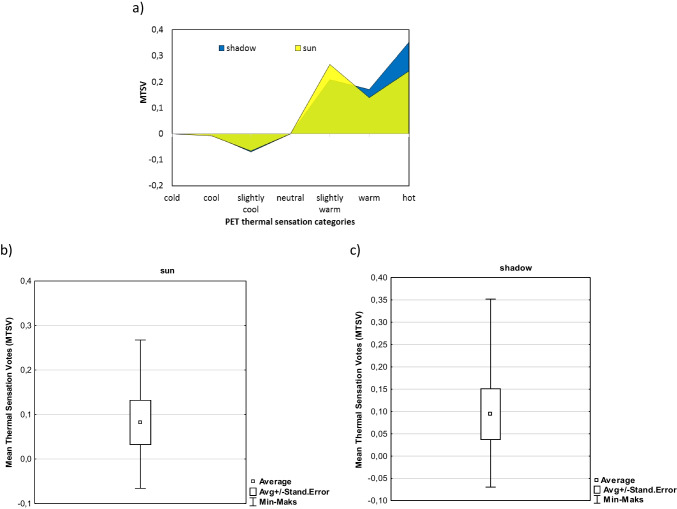
Recreationists’ in the sun and in the shade mean thermal sensation votes (MTSV) in reference to PET categories (**a**) and on the box plot with statistic characteristics (**b**)

They were two types of survey places: in the shade, most often in the natural shade of trees (64.8%), or in the open space, i.e., in the sun (35.2%), with direct sunlight. Mean thermal sensation votes (MTSV) were counted for both groups (in the shade and in the sun) of people in the ranges of PET index (Fig. 3). Thermal impressions of both groups of these people differed significantly (*t* = 6,469; *R*^2^ = 0.893; *p* = 0.001). People being in the shade tended to assess thermal conditions in a more extreme way than people in the sun, which indicate a more sensitive perception of extreme thermal conditions and a weaker adaptation to them. Their MTSV were higher in “warm” and “hot” PET thermal sensation categories. On the other hand, people in the sun more often felt the “slightly warm” PET thermal sensation category. Both groups had the same sensations for the “slightly cool” thermal sensation category.

## Thermal sensations and preferences

**Fig. 4:**
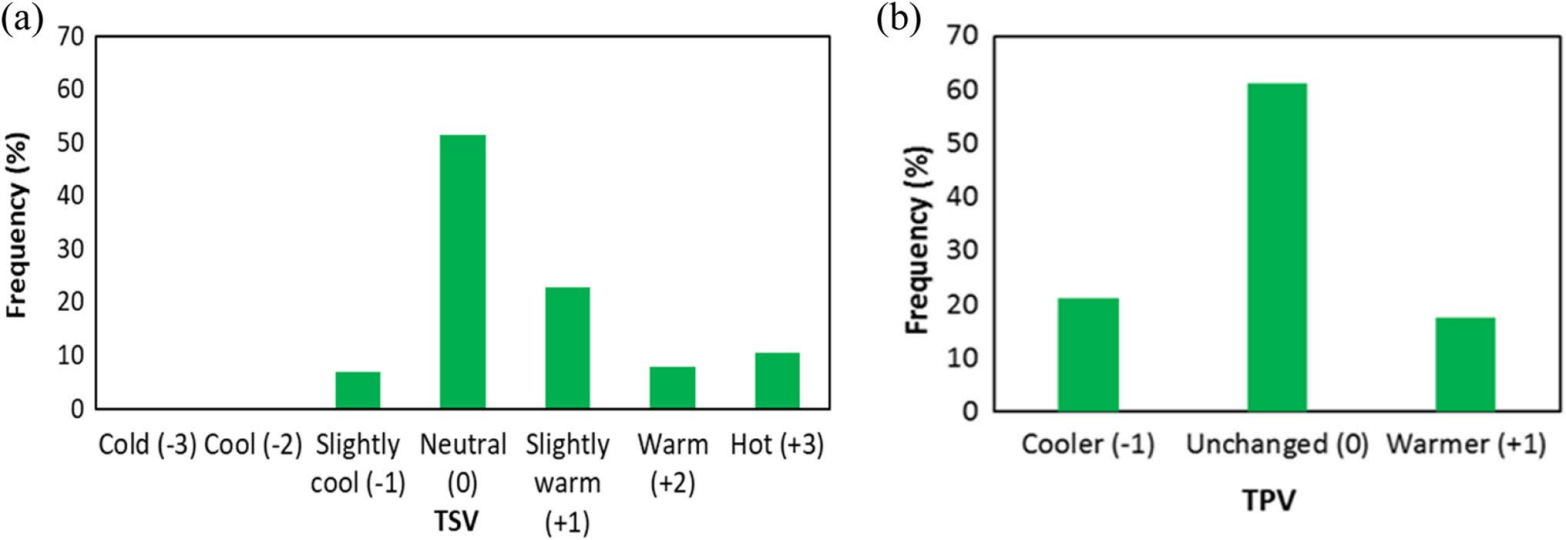
Percentage (%) of respondent’s votes for **a** thermal sensation votes (TSV) and **b** thermal preferences votes (TPV) during summer 2019 (*N* = 776)

Thermal sensation votes (TSV) of respondents varied over the summer, but the respondents felt “neutral” comfort most often (51.5%), next subcomfort, defined by a “slightly warm” feeling of 22.9%. On the other hand, the feeling of discomfort of “hot” (+ 3) and “warm” (+ 2) occurred only in 10.4% and 8.0%, respectively, and the least frequent feeling of “slightly cool” (− 1) 6.8% (Fig. 4a). In the case of thermal preferences (TPV), the vast majority of respondents (61.2%) expressed satisfaction with the air temperature (Fig. 4b), and the same accepted the thermal conditions and wanted them not change. A total of 21.3% of respondents preferred to be cooler (− 1), and slightly less (17.5%) of respondents would like it to be warmer (+ 1).

**Fig. 5:**
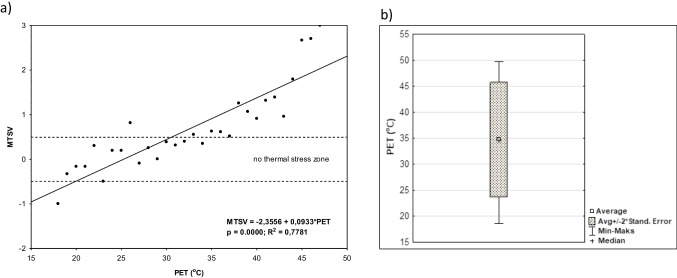
Mean thermal sensation votes (MTSV) compared PET variables (**a**). The range of PET variables for neutral sensations (TSV = 0) in summer versus PET variables (**b**)

The linear relationship between the biometeorological conditions determined by the PET index and the respondents’ thermal sensations was determined for the study (summer) period. A moderately strong correlation was found between these variables (MTSV and PET) with *R*^2^ = 0.778, *p* < 0.0001) (Fig. 5a). Linear equation gives the opportunity to determine the PET values assessed by recreationists in the park as neutral thermal zones. Assuming that the − 0.5 to + 0.5 range of MTSV corresponds to “no thermal stress” (Matzarakis et al. 1999), neutral temperature is considered as the temperature at which humans feel neither cold nor warm (Fanger 1972) and was defined by PET values of the range between 6.3 and 21.8 °C. The median of PET for neutral thermal sensations (TSV = 0; *N* = 776) was 32.5 °C for the summer (Fig. 5b).

**Fig. 6:**
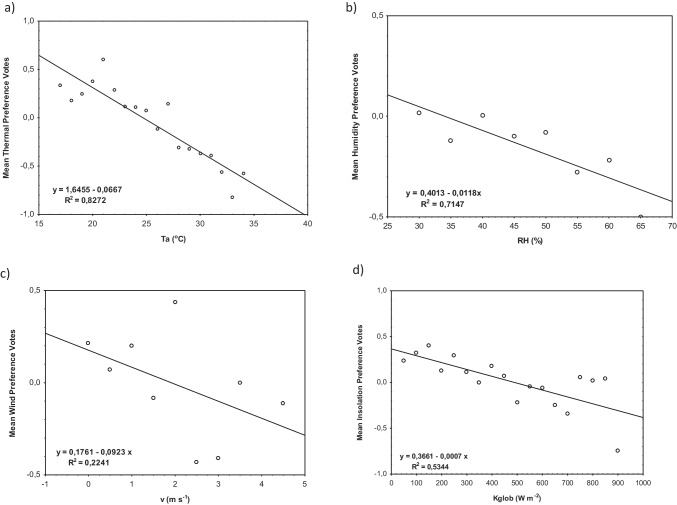
Mean preference votes (MPV) for meteorological parameters: **a** air temperature, **b** relative humidity, **c** wind speed, **d** global solar radiation

Then, respondents’ mean preference votes (MPV) were presented as a function of the respective meteorological parameters (Fig. 6). The relationship between the increase of Ta, v, Kglob, and RH and a preference for lower values parameters of weather was stated. These negative relationships were relatively strong, with *R*^2^ > 0.5, and statistically significant at *p* < 0.0001, except for the wind speed which the relationship was not statistically significant. The strongest correlation was found between Ta and thermal preferences (*R*^2^ = 0.827). When Ta values increased to 27 °C, the respondents’ preferences tended air temperature to be higher, and above this value, the respondents preferred a lower one. The option “no change” in air temperature, regardless of biothermal conditions, was mostly preferred. Similarly to the temperature, strong relationship was found between RH and humidity preferences (*R*^2^ = 0.715). When the RH values increased to 39%, the respondents indicated “no change” in the air, whereas above this value, the RH preference was lower air humidity, although in the analyzed period the option “no change” was preferred. In the case of solar radiation intensity, the dominant preference among the respondents was “no change,” while for radiation intensity up to 500 W∙m^−2^, the most frequently indicated option was an increase in the intensity, and above 500 W∙m^−2^ a decrease one. The worst and statistically insignificant relationship was obtained for wind speed and wind speed preferences (*R*^2^ = 0.224). The preference for “no change” in wind speed was dominant, but with low wind speed (v < 2 m∙s^−2^), higher wind speed (air movement) was preferred.

**Fig. 7:**
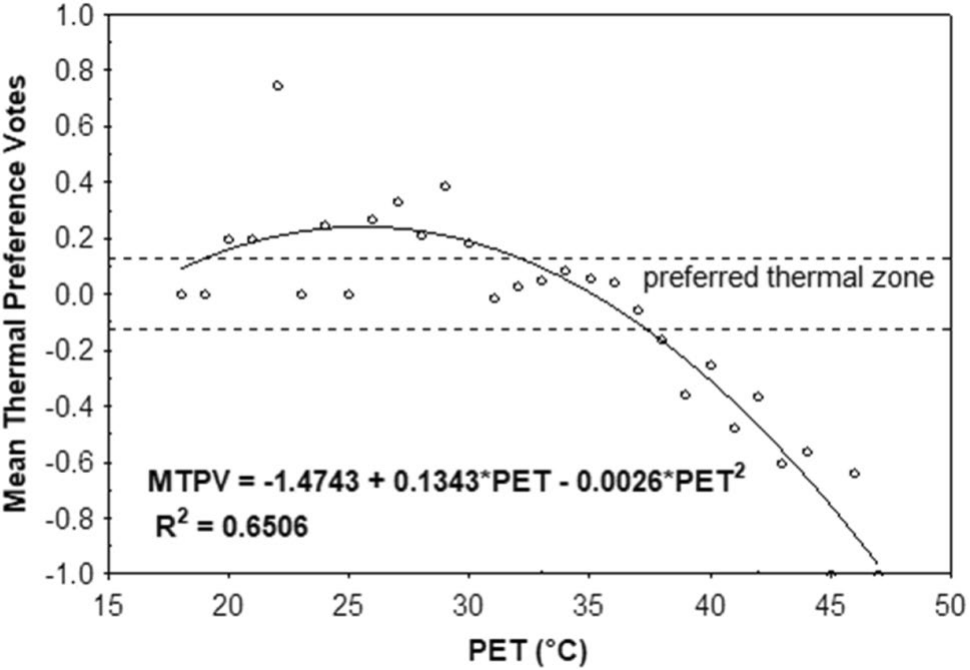
Mean thermal preferences votes (MTPV) in relation to PET variables at 1 °C in summer

In order to determine the optimum thermal zone for recreation and urban tourism during the summer, a binomial regression model for mean thermal preferences (MTPV) in PET intervals was calculated (Fig. 7). Using the procedure developed by Kovács et al. (2016), the preferred spectrum of thermal conditions for tourism and recreation in Warsaw can be related to the PET range between 27.3 and 31.7 °C. These values represent the upper limit of “slightly warm” and “warm” thermal sensations based on the PET scale for Central Europe, and they are much higher than the previously defined neutral thermal range.

**Fig. 8:**
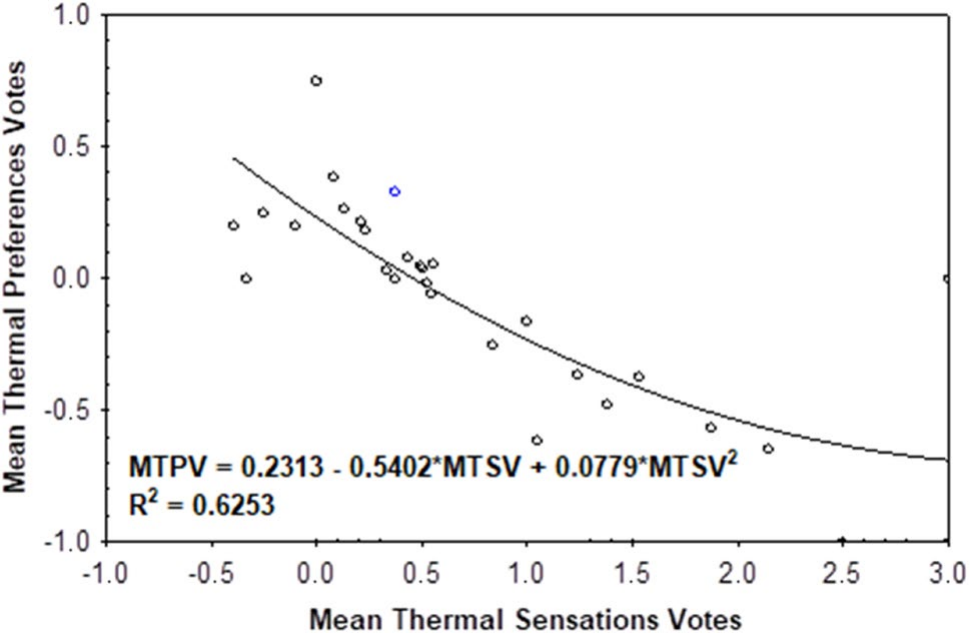
Respondents’ mean thermal preference votes in relation to mean thermal sensations votes in 1 °C PET ranges

Thermal sensations with their thermal preferences were also compared. Pearson’s correlation between TPV and TSV was negative and moderately strong for the summer period (*r* =  − 0.57, *N* = 776, *p* < 0.05). Analyzing the MTPV of respondents with their respective MTSVs, the preference was for slightly warmer than real conditions in most cases (Fig. 8). For MTSV less than or equal to 0 (neutral), the MTPVs were usually between 0.0 and 0.5, indicating a wish a slightly higher current air temperature. Assuming that MTPV = 0 is connected to a comfortable and desirable thermal environment, the preferred thermal sensation for recreationalists and tourists in the park is 0.5, which corresponds to a sensation between “neutral” and “slightly warm.”

## Research on the perception and weather preferences of the respondents

**Fig. 9:**
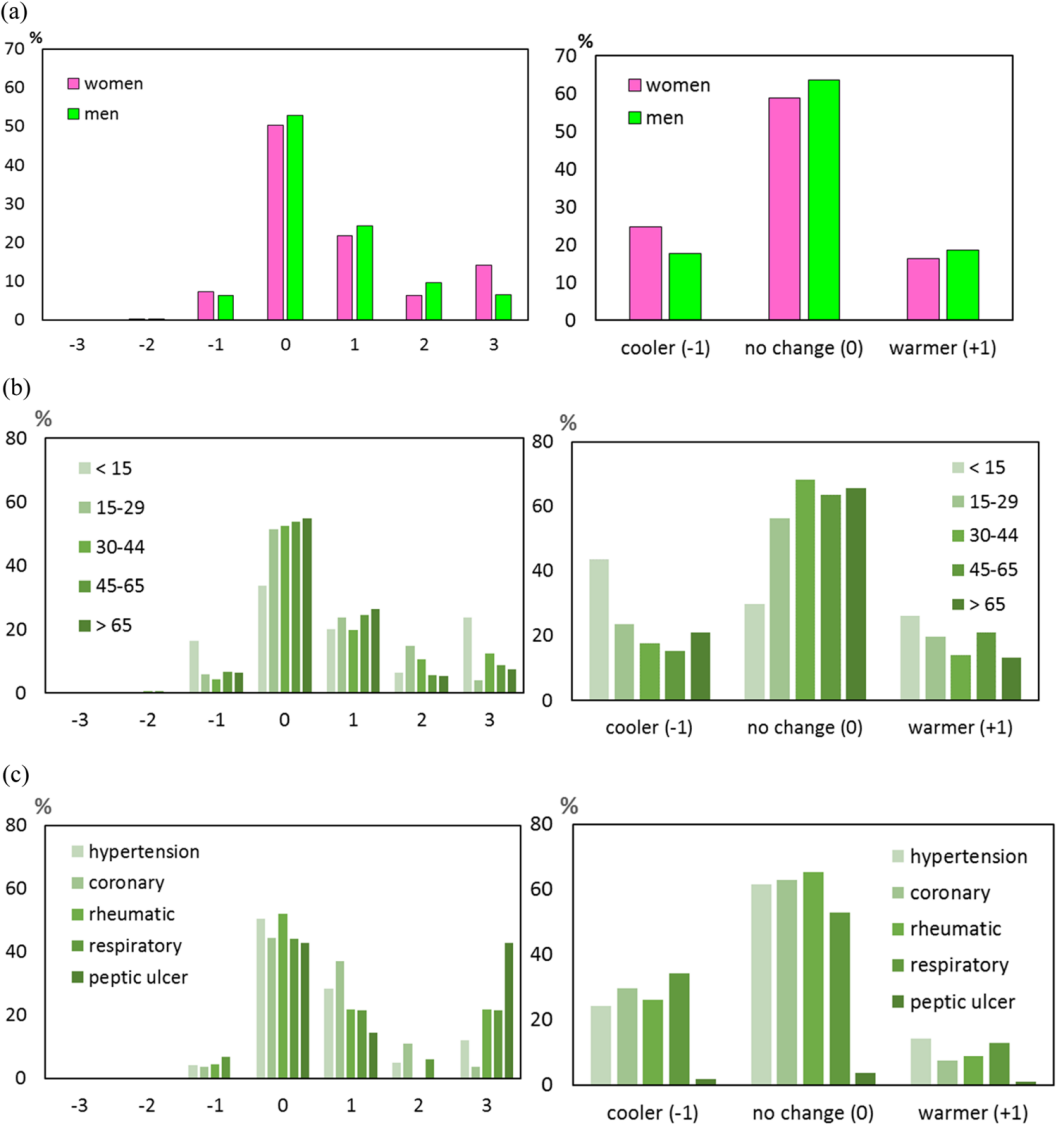
Percentage (%) votes on thermal sensation (TSV) and thermal preference votes (TPV) in relation to individual respondents’ characteristics: **a** gender, **b** age, **c** type of diseases. TSV: − 3—“cold,” − 2—“cool,” − 1—“slightly cool,” 0—“neutral,” + 1—“slightly warm,” + 2—“warm,” + 3—“hot”

Among the recreationists visiting the Powsin Culture Park, the men more than the women perceived the thermal conditions as “neutral” (0) and “slightly warm” (+ 1) and “warm” (+ 2). On the other hand, women were more sensitive than men to extreme thermal sensations, especially “hot” (+ 3). Men more often than women indicated greater acceptance for comfortable (no change) (0) and warmer (warmer) thermal conditions (+ 1), whereas women preferred cooler (− 1) thermal conditions and were more often dissatisfied with the air temperature during the summer (Fig. 9a). It was also shown that the relationship between MTSV and thermal sensations is very strong and statistically significant (*R*^2^ = 0.963, *p* < 0.05), while the relationship between MTPV and thermal sensations is statistically insignificant.

In the analyzed sample, no statistically significant correlation was found between the age of the respondents and their thermal sensations (Fig. 9b). All age groups indicated the highest frequency of neutral thermal sensation vote, which decreased with feeling more extreme thermal conditions. The group under 15 years of age was the exception, which showed the highest frequency of extreme conditions indications and, at the same time, the lowest resistance to extreme thermal sensations “hot” (+ 3). The elderly showed the highest resistance to the burdensome conditions of warm and hot thermal sensation. Taking into account the preferences, the analyzed sample showed an increase in the acceptance of conditions with age. Elder respondents in particular wanted warmer and cooler weather less frequently. On the other hand, the youngest respondents (below 15 age) most often wished for cooler weather.

The dependence type of disease on age as well as the relationship with the type of disease and thermal sensations and preferences were also examined. The respondents indicated an increase in the incidence of diseases with age most often. However, more people under 30 suffered from respiratory diseases (including allergies and asthma). Most respondents had hypertension and respiratory diseases, and proportions between men and women were similar (detailed data are not shown in the paper).

Age-related diseases occurrence except respiratory diseases have a similar distribution to the age structure and its thermal sensation shown in Fig. 9c. The only discrepancy can be seen in peptic ulcer disease, which shows a marked increase in frequency (42.9%) with extreme “hot” weather (+ 3).

In people indicating diseases, there is a higher frequency of preferences for neutral thermal conditions, and then for cooler ones. Mentioned statistical relationships between the type of disease and thermal sensation votes and preferences were weak and not significant.

## Discussion

This research assumes that people living in the city, staying outdoors, especially in green areas such as squares, parks, and forests for recreational purposes, perceive the thermal environment differently than people being outside for other purposes and in places highly urbanized, e.g., city center, pavements, and open urban space. An individual’s thermal perception cannot be fully explained by the human energy balance, because psychological and behavioral factors influence our thermal sensations and preferences towards weather elements (Spagnolo and de Dear 2003; Lin et al. 2011).

Moreover, people staying in a specific place to rest are usually in a good mood and feel the thermal conditions as more comfortable (Knez and Thorsson 2008; Yin et al. 2012). Thorsson et al. (2004) showed that due to the voluntary nature of outdoor recreation, people are more tolerant of different weather conditions. More often, they are more accurately assessed by passers-by or people staying in a given place out of necessity.

In this study, similarly to Lindner-Cendrowska and Błażejczyk (2018), neutral thermal conditions were determined by the use of linear regression and limitation by the range of − 0.5 to + 0.5 MTSV. This procedure resulted in a thermoneutral range defined by PET values from 6.3 to 21.8 °C. Such a wide thermoneutral range is a consequence of the following factors: a wide range of PET amplitude during the year in Poland and a small frequency of votes indicating the extreme thermal sensation of MTSV (90% of the values are between − 1.5 and + 1.5). The last factor can be explained by clothing insulation adjustments and the adaptation of respondents during the whole year. Krüger et al. (2013), using the same methodology, obtained a much narrower range (9–18 °C) of the neutral value for the warm half of the year in a maritime moderate climate. Because the neutral temperature values vary throughout the year, Kovács et al. (2016) suggest introducing an adjustment to the neutral PET thresholds for each season separately, which seems justified in this type of climate. It concerned, for example, in the research carried out by Lindner-Cendrowska and Błażejczyk (2018) for this climatic zone but for the city center.

Tourists, regardless of their origin, prefer sun and warm weather in their holiday or recreational destination (Lise and Tol 2002), and although having different types of activity, they indicate different preferences for meteorological elements (Bafaluy et al. 2014). Lindner-Cendrowska and Błażejczyk (2018) showed that in the urban environment of Warsaw (not in the park but inside the built area), people staying outdoors for tourism and recreation preferred warmer than current thermal conditions for most of the year even during the summer when PET index exceeded 29 °C (which corresponds to “warm thermal conditions”) and predominate satisfaction with thermal conditions. On the other hand, Kántor et al. (2012a, 2012b) claimed that humans are most vulnerable in their thermal perception to wind and insolation. According to Lindner-Cendrowska and Błażejczyk (2018) in the city of Warsaw, tourists in an urban environment preferred intensive solar radiation and slight wind velocity (less than 2 m·s^−1^). It can be partially explained by the small differentiation of wind conditions in a dense built-up area. In case of the urban park area, the presence of trees and shrubs gives to similar wind conditions. On the other hand, the same factor (the presence of trees) gives the larger areas of shadow and improves micrometeorological conditions to be more comfortable.

## Conclusions

During the field study period in the Culture Park in Powsin, potential equivalent temperature (PET) values varied from 18.6 °C (4th July) to 47.7 °C (26th June), reflecting the thermal sensation from neutral to hot. In the analyzed period, the following classes were dominant: “hot” (45.2%) and “warm” (41.0%), which together give the amount 86.2%, and only 10.4% were more pleasant thermal sensations—“slightly warm” and 3.4% “neutral.” In the case of thermal preferences (TPV), the vast majority of respondents (61.2%) expressed satisfaction with the air temperature, and the same accepted current thermal conditions and wanted them not to change. The preferred spectrum of thermal conditions in tourism and recreation in Warsaw can be related to the PET range between 27.3 and 31.7 °C. These values represent the upper limit of “slightly warm” and “warm” thermal sensations based on the PET scale for Central Europe, and they are higher than the previously defined neutral thermal range.

Air temperature of 27 °C and relative humidity of 39% seem key for weather perception during summer in the city park. When the temperature increased to 27 °C, the respondents’ preferences tended air temperature to be higher, and above this value, the respondents preferred lower one. When the relative humidity increased to 39%, the respondents indicated “no change,” whereas above this value, the preference was lower one or “no change.” Dividing the whole group of respondents into different categories, it was assumed that the thermal sensations for humans included to these categories would be different. In this aspect, the following relationships were found. Men more than the women perceived the thermal conditions as “neutral,” “slightly warm,” and “warm.” On the other hand, women were more sensitive than men to extreme thermal sensations, especially “hot.” Men more often than women indicated greater acceptance for comfortable and warmer thermal conditions, whereas women preferred cooler conditions and were more often dissatisfied with the air temperature. The analyzed sample showed an increase in the acceptance of conditions with age. Elder respondents in particular wanted warmer or cooler weather less frequently. On the other hand, the youngest respondents (below 15 age) most often wished for cooler weather.

In people indicating diseases, there is a higher frequency of preferences for neutral thermal conditions, and then for cooler ones, but detailed statistical relationships between the type of disease and thermal sensation votes and preferences were weak and not significant.

The presented results of the research show the distribution and analysis of thermal sensations in the city park in the summer—the best time for recreation. In order to evaluate the importance of green areas and city parks for urbanized areas and their inhabitants on the background city conditions, the same analysis should be carried out simultaneously in areas with different levels of building density (city center, urban residential district, etc.) and on a similar, representative sample of respondents using the same methods.

## Supplementary Information

Below is the link to the electronic supplementary material.

Supplementary file1 (PDF 559 KB)

## Data Availability

The data collected during this study are included in this published article. The data from respondents were collected in paper form. The datasets analyzed during the study are available from the authors on reasonable request.
