# Supplementary material for: Summer weather perception and preferences in Powsin Culture Park (Warsaw, Poland)
Source: Int J Biometeorol. 2023 Mar 27;67(5):793–805. doi: 10.1007/s00484-023-02455-x (PMC10167111; doi:10.1007/s00484-023-02455-x)
Supplement: Supplementary file 1 — Supplementary file1 (PDF 559 KB) [file 484_2023_2455_MOESM1_ESM.pdf]

Date:	**Weather perception questionnaire**	*Time:*

1. Air temperature:

- Being dressed as you are at the moment, how do you assess your current thermal sensation:

cold	*cool*	*slightly cool*	*neutral*	*slightly warm*	*warm*	*hot*
(-3)	*(-2)*	*(-1)*	*(0)*	*(+1)*	*(+2)*	*(+3)*

- Would you prefer it to be:

cooler	*unchanged*	*warmer*
(-1)	*(0)*	*(+1)*

2. Wind:

- Would you like the wind to blow:

weaker	*stay unchanged*	*harder*
(-1)	*(0)*	*(+1)*

3. Sun:

- Would you like the sun to shine:

less intensive	*stay unchanged*	*more intensive*
(-1)	*(0)*	*(+1)*

4. Cloudiness:

- Would you prefer now the sky to be:

cloudless	*covered with a few clouds*	*completely clouded*
(0)	*(1)*	*(2)*

5. Humidity:

- Would you like the humidity to be:

lower	*unchanged*	*higher*
(-1)	*(0)*	*(+1)*

6. Clothing:

- Choose the clothes you are wearing now:

T-shirt / top / shirt with short sleeves / shirt / blouse with long sleeves / vest / sweatshirt / jumper / cardigan / turtleneck /

shorts / short skirt / trousers / long skirt / ¾ trousers /

socks / leg wormers / tights / leggings /

scarf / gloves /

<u>outer layer:</u>
1-layer jacket / 1-layer trench coat / quilted jacket / woollen coat / down vest / leather jacket / fleece jacket / other overcoat (what?) ....

shoes / trainers / boots / ankle boots / Wellingtons / sandals

<u>headdress:</u> *yes / no*
hat / cap / head shawl / hood / headband /

– Does any of your clothing elements have special thermal properties (gore-tex, hydrotex, polartec etc.)? ....

7. Recent physical activity:

- Where have you been before coming here (last 30 minutes):

outdoors	*indoors (with / without air conditioning)*	*in the vehicle (with / without air conditioning)*	*other.............*

- During last 30 minutes your main activity was:

sitting	*standing*	*walking*	*other ....*

8. Reason of being in this place :

- Are you in this place for the tourist / recreational reason: *Yes / No*

9. Personal information:

- Age: *below 15 / 15-29 / 30-44 / 45-65 / over 65* - Sex: *male / female*
- Where do you live (country and city): ....
- For how long have you been in Warsaw or its surroundings (number of days): ....
- Do you suffer from any of diseases mentioned below:
  hypertension / coronary (heart) disease / rheumatic disease / respiratory system disease (including asthma and allergy) / chronic gastric ulcer disease /
